# Supplementary material for: Stimulus-specific adaptation to behaviorally-relevant sounds in awake rats
Source: PLoS One. 2020 Mar 25;15(3):e0221541. doi: 10.1371/journal.pone.0221541 (PMC7094827; doi:10.1371/journal.pone.0221541)
Supplement: S1 Fig — Three different characterizations are displayed. a. Oscillograms of the two words. The two high-amplitude vowels in each word are clearly visible. b. Average power spectrum in 1/3 octave bands. The average power spectrum has been carefully equalized between the two stimuli. c. Spectrograms of the two stimuli. The ladder-like structures are the harmonics of the pitch of the two vowels, set to 300 Hz. (PDF) [file pone.0221541.s001.pdf]

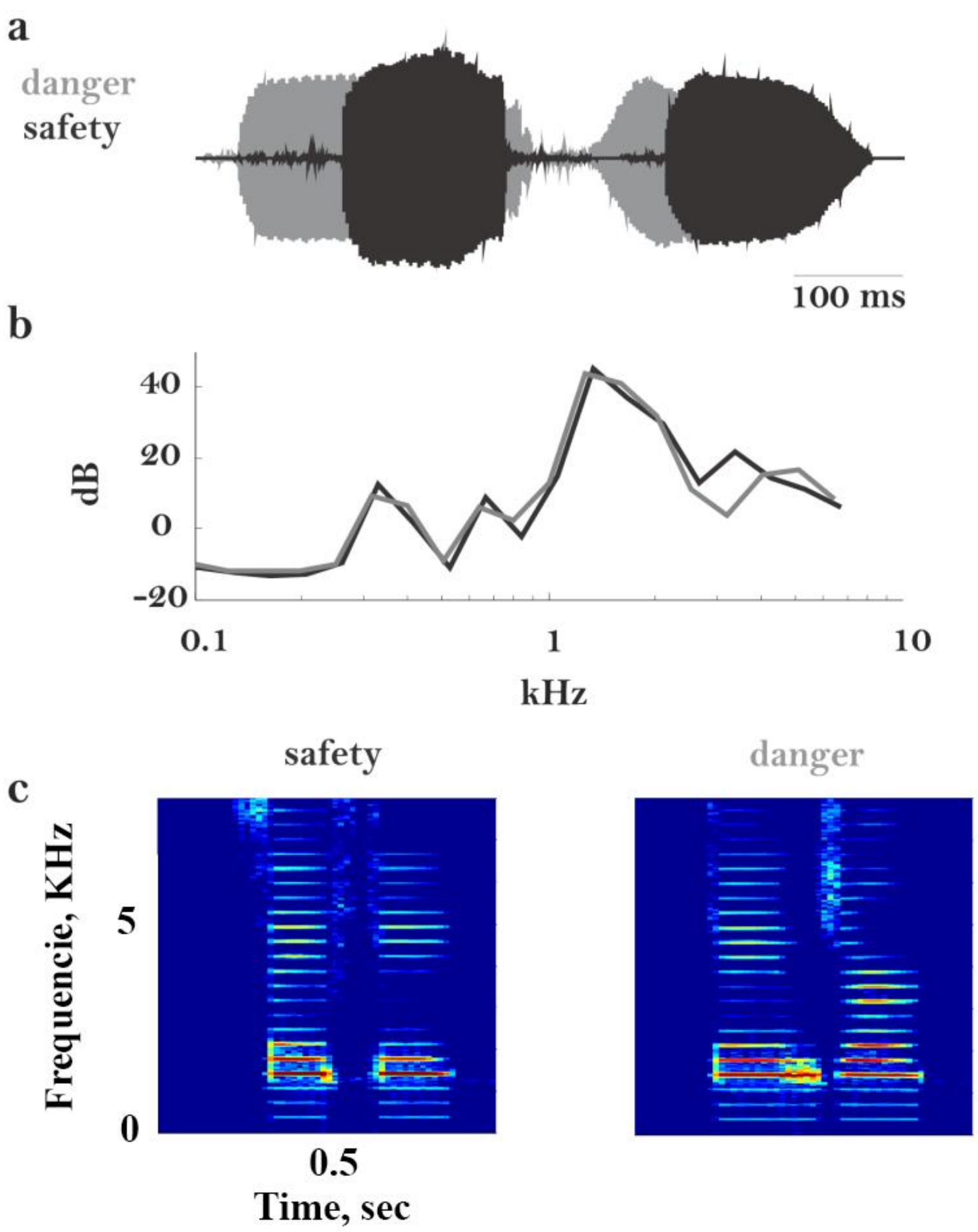

Supplementary Figure 1. The word-like stimuli.

Three different characterizations are displayed. a. Oscillograms of the two words. The two high-amplitude vowels in each word are clearly visible. b. Average power spectrum in 1/3 octave bands. The average power spectrum has been carefully equalized between the two stimuli. c. Spectrograms of the two stimuli. The ladder-like structures are the harmonics of the pitch of the two vowels, set to 300 Hz.
