# Supplementary material for: Stimulus-specific adaptation to behaviorally-relevant sounds in awake rats
Source: PLoS One. 2020 Mar 25;15(3):e0221541. doi: 10.1371/journal.pone.0221541 (PMC7094827; doi:10.1371/journal.pone.0221541)
Supplement: S2 Fig — The algorithm used the video movie of the test episode. Starting from the individual frames (a), pairwise differences between successive frames were computed (b). The number of non-zero pixels was counted and smoothed. Panel c (blue) shows an example of such a smoothed trace. Freezing periods were determined by thresholding this trace (red line). Panel c (black) shows the resulting decisions. The fraction of time that freezing episodes occupied was determined separately for a baseline period (gray rectangle), for presentations of the CS+ (red) and for presentations of the CS- (green), as shown in panel d. (PDF) [file pone.0221541.s002.pdf]

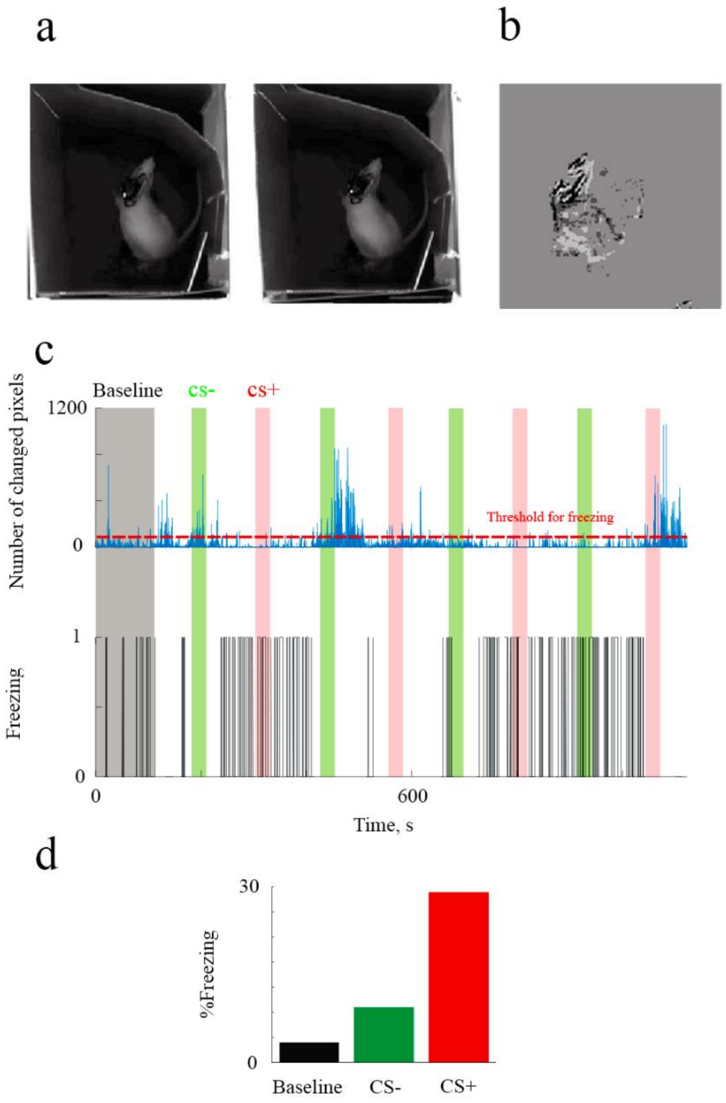

Supplementary Figure 2. Detection of freezing.

The algorithm used the video movie of the test episode. Starting from the individual frames (a), pairwise differences between successive frames were computed (b). The number of non-zero pixels was counted and smoothed. Panel c (blue) shows an example of such a smoothed trace. Freezing periods were determined by thresholding this trace (red line). Panel c (black) shows the resulting decisions. The fraction of time that freezing episodes occupied was determined separately for a baseline period (gray rectangle), for presentations of the CS+ (red) and for presentations of the CS- (green), as shown in panel d.
